# Predictive Role of Pre-Radiotherapy D-Dimer and Inflammatory Markers in Monitoring Outcomes After Treatment in Hormone-Positive Breast Cancer: A Retrospective Cohort Study

**DOI:** 10.3390/diagnostics16040582

**Published:** 2026-02-14

**Authors:** Kimia Cepni, Tugce Hilal Ucgun, Tugce Dursun Ucar, Bahar Cepni, Abdulkerim Uygur, Ebru Sen, Hilal Ozkaya, Huriye Senay Kiziltan

**Affiliations:** 1 Department of Radiation Oncology, Basaksehir Cam and Sakura City Hospital, University of Health Sciences, 34840 Istanbul, Türkiye; tuceucgun@gmail.com (T.H.U.); tugceucar364@gmail.com (T.D.U.); hskiziltan@gmail.com (H.S.K.); 2 Department of Radiation Oncology, Heidelberg University Hospital, 69120 Heidelberg, Germany; baharcepni@gmail.com; 3 Cerkezkoy District Health Diroctorate, 59500 Tekirdag, Türkiye; abdulkerimuygur@gmail.com; 4 Department of General Surgery, Basaksehir Cam and Sakura City Hospital, University of Health Sciences, 34840 Istanbul, Türkiye; ebrusenoran@windowslive.com; 5 Department of Family Medicine, Basaksehir Cam and Sakura City Hospital, University of Health Sciences, 34840 Istanbul, Türkiye; ozkaya2012@gmail.com

**Keywords:** radiotherapy, D-dimer, IL-6, lymphopenia, breast cancer, pre-RT D-dimer, HR-positive, ER/PR positive breast D-dimer, D-dimer thresholds

## Abstract

**Background/Objectives**: D-dimer, a fibrin degradation product, is associated with tumor growth and metastasis. In breast cancer, high concentrations of D-dimer are linked to more advanced disease stages and metastatic spread. This research aimed to examine the relevance of D-dimer levels in estrogen and progesterone hormone receptor (HR)-positive breast cancer. **Methods**: This retrospective single-center cohort study included patients with HR-positive breast carcinoma who underwent adjuvant or palliative radiotherapy in Türkiye. Pre- and post-radiotherapy blood test results, including D-dimer levels, were required. D-dimer, lymphocyte percentage, and interleukin-6 levels were measured for evaluation. All statistical analyses were performed using R software (version 4.4.2) to evaluate associations between D-dimer levels and other laboratory parameters. Univariate and multivariate Cox proportional hazards regression were performed to identify prognostic factors for progression-free survival (PFS) and overall survival (OS). Statistical significance was defined as *p* < 0.05. **Results**: Elevated D-dimer levels were associated with worse Eastern Cooperative Oncology Group performance status, advanced disease stages, metastasis, elevated IL-6 and CRP levels, and lower lymphocyte counts. Pre-RT D-dimer was a strong prognostic factor. Patients with D-dimer ≤ 0.3 µg/mL showed significantly superior OS and PFS (>60 months; *p* < 0.001), with only one event, and this remained significant in multivariate analysis (OS: HR 4.55, 95% CI 1.89–11.3; *p* = 0.002; PFS: HR 3.43, 95% CI 1.54–7.8; *p* = 0.004). Similarly, D-dimer ≤ 0.5 µg/mL was associated with improved OS (4/72 vs. 19/40 events; *p* < 0.001) and longer PFS, confirmed in multivariate analysis (OS: HR 4.37, 95% CI 1.72–9.86; *p* = 0.002; PFS: HR 3.88, 95% CI 1.67–9.1; *p* = 0.003), whereas levels > 0.5 µg/mL predicted worse outcomes. Using a 0.65 µg/mL cutoff, patients with D-dimer > 0.65 µg/mL had significantly shorter OS (median 25.5 months; 95% CI, 18–NA) compared with those ≤0.65 µg/mL (median not reached; *p* < 0.001), and this remained independently significant (OS: HR 5.10, 95% CI 1.9–13.6; *p* < 0.001; PFS: HR 4.68, 95% CI 1.83–11.9; *p* = 0.002). **Conclusions**: D-dimer is an accessible, non-invasive biomarker with predictive and prognostic significance in HR-positive breast cancer. Elevated D-dimer levels are suggestive of a more aggressive disease and poorer survival outcomes. This has the potential to facilitate early assessment of treatment efficacy and disease progression. This study has several limitations. Its retrospective, single-center design may introduce selection bias and limit generalizability. Although the sample size was sufficient to detect significant associations, validation in larger, multi-center cohorts is warranted to confirm the prognostic value of D-dimer.

## 1. Introduction

Breast cancer remains one of the leading causes of death among women aged 20 to 59, with various factors influencing its prognosis. Although mortality rates decreased by 44% between 1989 and 2022, early diagnosis and the development of new laboratory biomarkers are crucial for improving survival outcomes [1].

Breast cancer shows considerable tumor heterogeneity, making it essential to evaluate key clinicopathological factors such as TNM stage, hormone receptor status, HER2 expression, and the presence of metastases to better understand and predict its diverse prognostic outcomes [2]. Other important factors are tumor grade, the presence of lymphovascular invasion, patient age, and ethnicity. Some biological markers, such as estrogen receptor/progesterone receptor status (ER/PR), HER2 expression, plasminogen activator and its inhibitor (PAI-1), serve not only as indicators of prognosis but also as predictors of treatment response. Tumor-induced blood clotting is closely linked to both the development and progression of cancer. In some cases, signs of venous thromboembolism can appear years before any obvious clinical symptoms of the tumor become apparent [3].

Studies suggest that the interaction between angiogenesis and hemostasis may play a role in promoting metastasis in breast cancer. Elevated plasma D-dimer levels are thought to reflect active remodeling of the tumor extracellular matrix [4].

D-dimer is formed when plasmin degrades cross-linked fibrin during the process of fibrinolysis. In clinical practice, D-dimer testing is commonly used to diagnose thromboembolic conditions such as deep vein thrombosis and pulmonary embolism [5].

Elevated plasma D-dimer levels reflect ongoing fibrin turnover and have been consistently associated with tumor burden, metastatic potential, and adverse prognosis in patients with breast cancer, supporting their role as a biomarker of tumor-driven coagulation activation rather than thrombosis alone [6].

In metastatic breast cancer, elevated D-dimer levels have been implicated as indicators of hypercoagulability, correlating with accelerated disease progression and reduced OS [7].

Moreover, the integration of D-dimer levels into prognostic models could significantly enhance personalized treatment strategies for breast cancer patients, particularly in high-risk cohorts, suggesting that these biomarkers could help stratify patients based on their likelihood of relapse and guide more tailored therapeutic interventions [8].

Although further investigation is required, D-dimer holds potential as a diagnostic instrument for cancer screening, tracking recurrences, and evaluating prognostic outcomes. It represents a non-invasive and cost-effective assessment that may improve early identification and therapeutic approaches for cancer patients [7].

In HR-positive breast cancer, it is important to distinguish the prognostic versus predictive intent of D-dimer. As a prognostic biomarker, elevated baseline D-dimer appears to reflect a more aggressive tumor–host interaction and is associated with inferior survival and higher relapse risk, independent of treatment. From a predictive perspective, D-dimer may help identify patients within HR-positive disease who are more likely to benefit from treatment intensification, closer surveillance, or tailored systemic strategies beyond standard endocrine therapy. While the current findings primarily support a prognostic role, the potential predictive implication of D-dimer in guiding risk-adapted management in HR-positive breast cancer warrants further prospective validation [1,3,4].

Prognosis in ER/PR-positive breast cancer is generally more favorable than in other types of breast cancer, yet studies on the role of D-dimer remain limited. This study analyzed the association between D-dimer levels and various tumor stages, disease progression, their prognostic significance and the efficacy of RT. Inhomogeneous findings were reported in the literature regarding the prognostic features of D-dimer in HR-positive breast cancer. We aimed to demonstrate that the D-dimer value can also serve as a guide for treatment decisions in breast cancer patients who underwent RT.

The inclusion of both adjuvant and palliative radiotherapy populations was intentional, as the primary aim of the study was to evaluate the biological and prognostic relevance of baseline D-dimer levels independent of treatment intent. D-dimer reflects systemic tumor–host interactions, coagulation activation, and overall disease burden—mechanisms that are not restricted to a specific treatment setting. Therefore, assessing its prognostic value across the disease spectrum provides insight into its potential robustness as a global risk-stratification biomarker.

## 2. Materials and Methods

### 2.1. Study Design and Patient Population

This single-center retrospective cohort study included 112 patients diagnosed with histologically confirmed ER and PR hormone receptor-positive invasive ductal breast carcinoma who underwent adjuvant or palliative RT at our institution between 2020 and 2024.

This study was conducted at a tertiary-care academic oncology center that serves as a regional referral hospital for breast cancer management. The institution manages both early-stage and advanced breast cancer patients and provides multidisciplinary oncologic care, including systemic therapy and RT.

Patients were included if they were 18 years or older, had complete clinical and treatment records and had available pre- and post-RT blood test results, including D-dimer levels. Patients with known active thrombosis, cardiac events or inflammation at the time of measurement were excluded from this study. Patients with HER2-positive or triple-negative and multiple metastatic disease were also excluded. ER/PR-positive patients with HER2 positivity were also excluded from the study. Patients labeled as “metastatic” were limited exclusively to brain- and bone-only oligometastatic disease, with no visceral or extracranial organ involvement, whose inflammatory profile would not lead to information uncertainty. Among 623 screened breast cancer patients, 454 were excluded due to unavailable D-dimer measurements or the presence of thrombosis, cardiac events, or inflammatory conditions. An additional 57 patients were excluded due to triple-negative or HER2-negative status or the presence of extensive metastatic disease (total excluded: 511).

Demographic and clinical characteristics were recorded, including age, comorbidities, ECOG performance status, TNM stage at diagnosis, presence and site of metastases (bone, brain, or none), and hormone receptor (ER/PR) expression status. Treatment details included RT target site (breast, bone, or brain). Data were also collected regarding the administration of chemotherapy (CT).

Median follow-up time was 36 months (3 to 60). At the time of analysis, 88 patients (78.5%) were alive.

### 2.2. Blood Sample Collection and Laboratory Analysis

Peripheral blood samples were collected before and after RT. The following parameters were measured: hematocrit, lymphocyte percentage, platelet count, C-reactive protein (CRP), ferritin, interleukin-6 (IL-6), fibrinogen, international normalized ratio (INR), and D-dimer. Laboratory analyses were performed in a standardized manner at our institutional laboratory, with D-dimer levels reported in µg/mL. Baseline D-dimer was measured within 7 days prior to the initiation of radiotherapy and was considered the primary exposure variable. Outcomes (overall survival and progression-free survival) were calculated from the start date of radiotherapy to the date of event (death or progression) or last follow-up. Post-radiotherapy D-dimer measurements were not used to define exposure and were analyzed separately for exploratory purposes, thereby preserving the temporal relationship between baseline biomarker assessment and clinical outcomes.

### 2.3. Statistical Analysis

Statistical analyses were conducted using R software (version 4.4.2). Variable normality was assessed through Kolmogorov–Smirnov and Shapiro–Wilk tests, along with Q-Q plots and histograms. Median (minimum–maximum) and mean ± standard deviation were used to express continuous variables, while frequencies (percentages) represented categorical variables. Mann–Whitney U and Kruskal–Wallis tests were applied for independent continuous variables, and the Wilcoxon signed-rank test was used for dependent variables.

#### 2.3.1. Survival Analysis

Relationships between continuous variables were analyzed using Spearman’s correlation test. Log-rank Kaplan–Meier analysis was utilized to compare overall survival and progression-free survival rates.

#### 2.3.2. Cox Proportional Hazards Regression Analysis

Univariate Cox regression analysis evaluated factors affecting overall survival and progression risk, with significant variables from this analysis included in the multivariate Cox regression analysis if the assumptions of Cox regression were met. Partial likelihood ratio and AIC values were examined for model fit assessment. Statistical significance was determined at *p* < 0.05.

### 2.4. Ethical Approval

The research protocol was reviewed and approved by the Ethics Committee of Basaksehir Cam and Sakura City Hospital (approval no. [2023.05.177]). As this was a retrospective study using anonymized patient data, informed consent was waived.

## 3. Results

### 3.1. Patient and Treatment Characteristics

**Figure 1:**
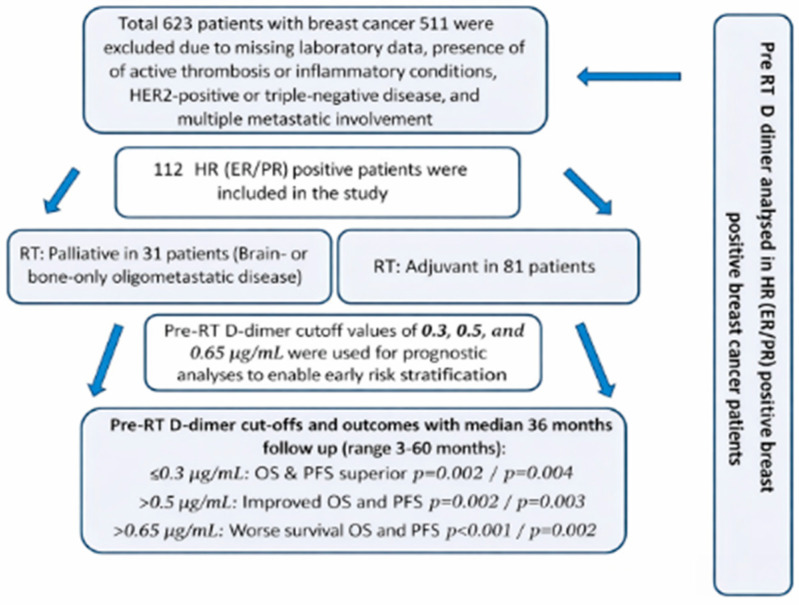
Flow diagram of study.

A total of 112 patients with ER- and PR-positive invasive ductal breast carcinoma were included in this study. The median age was 51 years (range: 38–88), and 14% of patients had comorbidities. A total of 81 patients (72%) were diagnosed with stage I/II/III disease, while 31 patients (27.6%) presented with stage IV disease. Metastatic sites included bone (20%) and brain (6.3%) metastases. The majority of patients (85%) had an ECOG performance status ≤ 2. ER/PR receptor expression levels were classified as high (70–100%) in 62% of patients, moderate (30–60%) in 18%, and low (10–20%) in 21%. With respect to treatment, 83 patients (74.1%) received CT during the course of their management. (Table 1). All 112 patients received hormone therapy (HT). Median follow-up time was 36 months (3 to 60). At the time of analysis, 88 patients (78.5%) were alive. The study flow diagram is presented in Figure 1.

Oligometastatic disease was operationally defined as the presence of ≤5 metastatic lesions confined to bone and/or brain, with no visceral organ involvement, confirmed on staging imaging (CT, MRI, and/or PET-CT).

#### 3.1.1. Radiotherapy

RT was primarily delivered to the breast/chest wall in 81 patients (72.3) and to metastatic sites in 31 patients (27.6%) (Table 2).

The median number of fractions was 25 (range: 2–30).

Adjuvant RT was administered to the majority of patients (81, 72.3%), who received a total dose of 4250–6000 cGy delivered in 15–30 fractions. Palliative RT was administered to 31 patients (27.6%), with prescribed doses ranging from 1600 to 4000 cGy delivered in 2–16 fractions.

Regarding fractionation, most patients received conventional fraction doses of 200–270 cGy (*n* = 88, 77.5%), while hypofractionated or high-dose-per-fraction schedules (250–900 cGy) were used in 24 patients (21.4%).

In terms of treatment sites, RT was most commonly delivered to the breast/chest wall (*n* = 81, 72.3%), followed by bone (*n* = 22, 19.6%) and brain metastases (*n* = 9, 8%).

##### Radiotherapy Technique

Intensity-modulated radiotherapy (IMRT) and volumetric modulated arc therapy (VMAT) were delivered using linear accelerator (LINAC)-based systems. IMRT allows highly conformal dose distribution through modulation of beam intensity according to tumor shape. VMAT was planned using arc therapy with single or multiple isocenters, enabling efficient dose delivery and reduced radiation-related toxicity, particularly in patients with large tumor volumes. Treatments were performed using Elekta Versa HD (Stockholm, Sweden) or TomoTherapy Radixact (Accuray Inc., Sunnyvale, CA, USA). Helical arc radiotherapy was delivered with continuous gantry rotation and simultaneous couch movement. TomoTherapy Radixact provides three-dimensional image-guided radiotherapy and represents an advanced form of IMRT.

Seventy-five patients (66.96%) received RT with helical arc IMRT and Tomotherapy, and 37 patients (33.04%) received RT with VMAT and Elekta Versa HD.

#### 3.1.2. Pre RT Dimer Levels

D-dimer was intentionally measured prior to RT within 7 days to avoid potential confounding from radiation-induced coagulation or inflammatory changes. Because the study aimed to evaluate the prognostic rather than treatment-response role of baseline D-dimer, this was checked during a follow-up examination one month after the radiotherapy session. D-dimer levels were measured in the institutional central laboratory using a quantitative immunoturbidimetric assay ([manufacturer, analyzer model]). The assay was calibrated according to the manufacturer’s standards, and internal quality controls were performed routinely. The results are reported in µg/mL FEU. The analytical measurement range and reference limits were consistent with laboratory accreditation standards.

The median pre RT D-dimer level was 0.2 µg/mL in stage I patients. The median D-dimer level was 1.2 µg/mL in stage IV patients. The median D-dimer level was 0.36 µg/mL (range: 0.10–14.00) before RT and 0.30 ng/mL (range: 0.10–16.00) after RT, with no statistically significant difference between pre- and post-RT values (*p* = 0.076) (Table 3).

Pre-RT D-dimer levels were significantly associated with several clinical parameters. Higher pre-RT D-dimer levels were observed in patients with bone and brain metastases compared to those without metastases (*p* < 0.001), stage IV disease (*p* < 0.001), ECOG > 2 (*p* < 0.001), and low/moderate ER/PR receptor levels (*p* = 0.011).

Patients who received RT to bone or brain sites (*p* < 0.001), higher RT fraction doses (250–900 cGy, *p* < 0.001), and lower total RT doses (2500–4250 cGy, *p* < 0.001) had significantly elevated pre-RT D-dimer levels.

Patients who underwent CT showed higher pre-RT D-dimer levels compared to those who did not (*p* = 0.043).

Post-RT D-dimer levels were significantly associated with metastasis sites, disease stage, ECOG status, ER/PR receptor expression, RT location (all *p* < 0.001), and CT use (*p* = 0.019).

Non-survivors had markedly higher D-dimer levels at both pre- and post-RT assessments compared to survivors (*p* < 0.001) (Table 4).

The correlations between clinical parameters and D-dimer levels before and after RT are presented in Table 5.

Overall survival (OS) was defined as the time from the start date of radiotherapy to death from any cause. Patients alive at the last follow-up were censored at the date of last clinical contact. Progression-free survival (PFS) was defined as the time from the start date of radiotherapy to the first documented disease progression or death from any cause, whichever occurred first. Patients without progression were censored at the date of the last radiologic or clinical assessment.

The median OS was 46 months (range: 5–60), and the median PFS was 45 months (range: 3–60) in all patients.

Patients with pre-RT D-dimer levels ≤ 0.3 µg/mL demonstrated significantly superior OS and PFS (>60 months; *p* < 0.001), with one event in the low-D-dimer group. (Figure 1). Similarly, patients with D-dimer ≤ 0.5 µg/mL had significantly superior OS (*p* < 0.001), with four events among 72 patients versus 19 events among 40 patients in the >0.5 µg/mL group. Elevated D-dimer levels (>0.5 µg/mL) were associated with substantially reduced PFS (*p* < 0.001).

**Figure 2:**
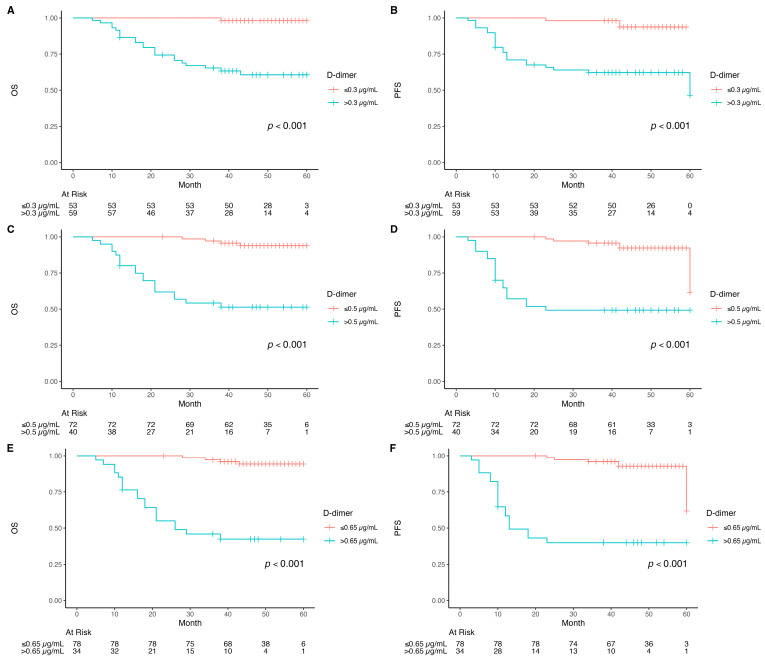
Kaplan–Meier curves of overall survival (OS) and progression-free survival (PFS). (**A**) OS when D-dimer > 0.3 µg/mL or ≤0.3 µg/mL; (**B**). PFS when D-dimer > 0.3 µg/mL or ≤0.3 µg/mL; (**C**) OS when D-dimer > 0.5 µg/mL or ≤0.5 µg/mL; (**D**) PFS when D-dimer > 0.5 µg/mL or ≤0.5 µg/mL; (**E**) OS when D-dimer > 0.65 µg/mL or ≤0.65 µg/mL; (**F**) PFS when D-dimer > 0.65 µg/mL or ≤0.65 µg/mL).

Patients with pre-RT D-dimer > 0.65 µg/mL had significantly shorter OS (median 25.5 months; 95% CI, 18-NA) compared to those with levels ≤ 0.65 µg/mL, who did not reach median survival (*p* < 0.001). Similarly, for PFS, patients with D-dimer < 0.65 µg/mL had a median of 12.5 months (95% CI, 12-NA) compared to the lower D-dimer group, which did not reach median PFS (*p* < 0.001) (Figure 2).

The 5-year OS and PFS rates were 99% and 97% for patients with D-dimer ≤ 0.3 µg/mL, compared to 56% and 55% for those with D-dimer > 0.3 µg/mL. Using a value of 0.5 µg/mL, 5-year OS and PFS were 97% and 96% versus 50% and 47%, respectively. At a level of 0.65 µg/mL, patients with lower D-dimer levels had OS and PFS of 96% and 95%, whereas those with higher levels showed rates of 46% and 45%.

**Figure 3:**
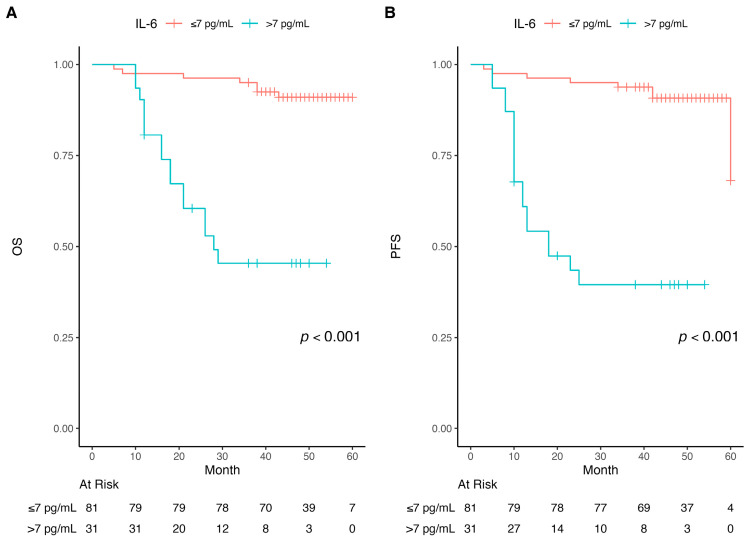
Kaplan–Meier curves of overall survival (OS) and progression-free survival (PFS). (**A**) Comparing OS for different pre-RT IL-6; (**B**) Comparing PFS for different pre-RT IL-6.

IL-6 levels similarly showed prognostic significance, with patients with IL-6 > 7 pg/mL demonstrating decreased OS (*p* < 0.001) and PFS (median 27 and 19 months, respectively) compared to those with IL-6 ≤ 7 pg/mL (median 60 and 60 months, respectively) (*p* < 0.001) (Figure 3).

Several negative prognostic factors were identified for OS, including stage IV disease (HR = 14.3, *p* < 0.001), ECOG > 2 (HR = 9.11, *p* < 0.001), bone (HR = 12.1, *p* < 0.001) and brain metastases (HR = 13.8, *p* < 0.001), IL-6 > 7 pg/mL (HR = 9.39, *p* < 0.001), and elevated pre-RT D-dimer levels: >0.3 µg/mL (HR= 6.13, *p* = 0.001), >0.5 µg/mL (HR = 8.45, *p* = 0.002), and >0.65 µg/mL (HR = 10.6, *p* < 0.001).

**Figure 4:**
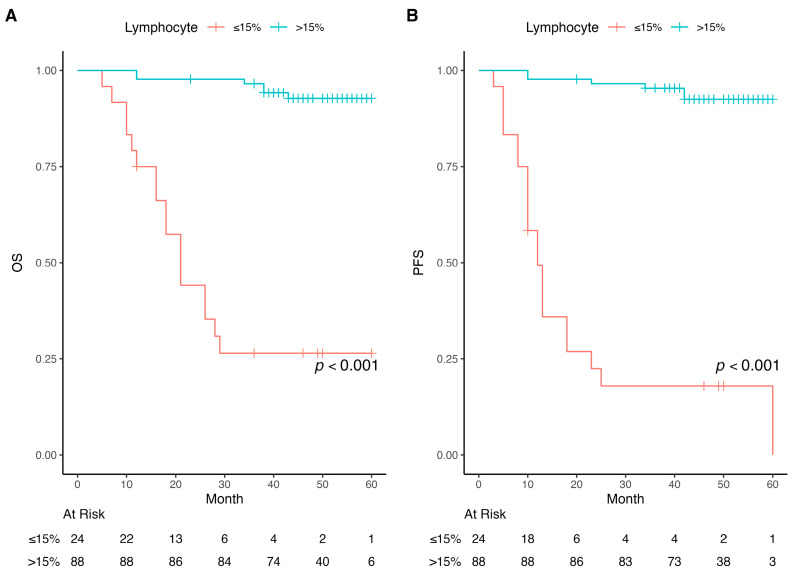
Kaplan–Meier curves of overall survival (OS) and progression-free survival (PFS). (**A**) Comparing OS for different pre-RT lymphocyte percentages; (**B**) Comparing PFS for different pre-RT lymphocyte percentages.

Protective factors for OS included high ER/PR expression (HR = 0.27, *p* < 0.001), lymphocyte percentage > 15% (HR = 0.05, *p* < 0.001) (Figure 4), and adjuvant RT in postoperative patients (HR = 0.15, *p* < 0.001).

In multivariate analysis, pre-RT D-dimer > 0.3 µg/mL remained an independent predictor of worse OS (HR = 4.55, *p* = 0.002), along with ECOG > 2 (HR = 5.21, *p* = 0.009) and lymphocyte percentage > 15% (HR = 0.08, *p* < 0.001) (Table 6). For overall survival (OS), both threshold- and low-level-based analyses consistently demonstrated the prognostic impact of pre-RT D-dimer. Patients with lower D-dimer levels (≤0.3, ≤0.5, and ≤0.65 µg/mL) had significantly superior survival, whereas increasing D-dimer levels were associated with progressively worse OS. In threshold-based analysis, pre-RT D-dimer > 0.3, >0.5, and >0.65 µg/mL were each significantly associated with inferior OS in univariate analysis (HR 6.13, 95% CI 2.45–12.26; *p* < 0.001; HR 8.45, 95% CI 3.21–19.73; *p* < 0.001; and HR 10.6, 95% CI 3.9–28.7; *p* < 0.001, respectively), and these associations remained independently significant in multivariate analysis (HR 4.55, 95% CI 1.89–11.3; *p* = 0.002; HR 4.37, 95% CI 1.72–9.86; *p* = 0.002; and HR 5.10, 95% CI 1.9–13.6; *p* < 0.001, respectively).

For progression-free survival (PFS), both threshold- and low-level-based analyses consistently confirmed the prognostic significance of pre-RT D-dimer. Patients with lower D-dimer levels (≤0.3, ≤0.5, and ≤0.65 µg/mL) demonstrated significantly improved PFS, whereas increasing D-dimer levels were associated with progressively poorer outcomes. In threshold-based analysis, pre-RT D-dimers of > 0.3, >0.5, and >0.65 µg/mL were each significantly associated with inferior PFS in univariate analysis (HR 4.90, 95% CI 2.32–10.17; *p* < 0.001; HR 6.50, 95% CI 2.9–14.6; *p* < 0.001; and HR 8.95, 95% CI 3.62–22.1; *p* < 0.001, respectively), and these associations remained independently significant in multivariate analysis (HR 3.43, 95% CI 1.54–7.8; *p* = 0.004; HR 3.88, 95% CI 1.67–9.1; *p* = 0.003; and HR 4.68, 95% CI 1.83–11.9; *p* = 0.002, respectively).

In patients who were given CT, PFS was significantly increased (HR = 0.5, *p* = 0.049). In multivariate analysis, ECOG > 2 (HR = 5.21, *p* = 0.009) and lymphocyte percentage > 15% (HR = 0.08, *p* < 0.001) (Figure 3) were independent predictors, and D-dimer > 0.65 µg/mL showed a significant trend (HR = 5.10, *p* < 0.001) (Table 7).

**Figure 5:**
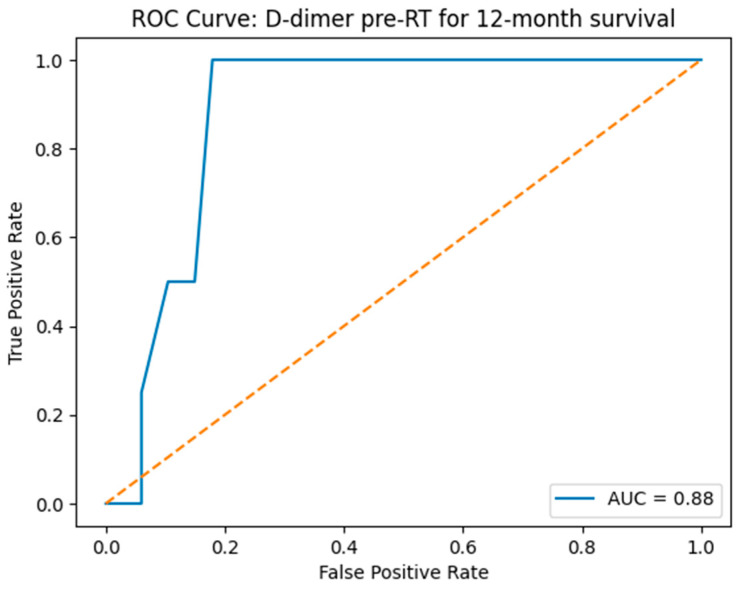
ROC curve for predictive performance of pre-RT D-dimer levels for 12-month survival. The dashed line represents the reference line (AUC = 0.5), indicating no discrimination.

Receiver operating characteristic (ROC) curve analysis was performed to evaluate the predictive performance of pre-RT D-dimer levels. ROC curve analysis demonstrated that pre-RT D-dimer levels had a strong ability to predict survival, with an area under the curve (AUC) of 0.88 (Figure 5).

The D-dimer cutoff of 1.21 µg/mL, derived from the ROC curve, yields a sensitivity of 100% and specificity of 87%. For a D-dimer value of 0.3 µg/mL, the sensitivity is 100% with 46% specificity. Similarly, the values of 0.5 µg/mL and 0.65 µg/mL both achieve 100% sensitivity, paired with specificities of 67.2% and 75%, respectively.

To clarify the analytic strategy, the 0.65 µg/mL cutoff was prespecified as the primary threshold based on its balanced prognostic discrimination and clinical relevance. The lower cutoffs (0.3 and 0.5 µg/mL) were analyzed as secondary/sensitivity thresholds to explore early risk stratification, while the ROC-derived value (1.21 µg/mL) was reported as exploratory.

Although the ROC analysis identified 1.21 µg/mL as the statistically optimal cutoff, this threshold was not selected for the primary prognostic analysis. One of the main objectives of the study was early risk identification, and lower D-dimer thresholds allowed the detection of prognostic separation at earlier stages of biomarker elevation. In our cohort, only a limited number of patients had D-dimer levels above 1.21 µg/mL, reducing statistical robustness and clinical applicability. In contrast, the lower cutoffs (0.3, 0.5, and 0.65 µg/mL) preserved 100% sensitivity while enabling earlier and more clinically meaningful risk stratification across a larger proportion of patients. For completeness, the ROC-derived cutoff and its diagnostic characteristics are reported in the Results section.

Subgroup analyses were performed to demonstrate the relationship between ER/PR and D-dimer levels and OS and PFS. The results of these analyses are shown in Table 8 and Table 9.

Patients with low ER/PR expression (<20%) demonstrated significantly higher pre-RT D-dimer levels and significantly shorter OS compared with those with high ER/PR expression (>70%). Median overall survival was 27.5 months in the ER/PR < 20% group versus 49.5 months in the ER/PR > 70% group (Mann–Whitney U test, *p* = 0.002) (Table 8).

Patients with low ER/PR expression (<20%) had significantly shorter PFS compared with those with high ER/PR expression (>70%). Median PFS was 23 months in the ER/PR < 20% group versus 49.5 months in the ER/PR > 70% group (Mann–Whitney U test, *p* = 0.002) (Table 9).

##### Treatment-Related Toxicity

Toxicity was evaluated using CTCAE v6.0. Grade 2 hematologic toxicity was observed in 58 patients (51.7%), including grade 3 toxicity in six patients (5.3%) during the RT. These patients were patients who had previously received CT. No grade 4 toxicity was observed.

## 4. Discussion

Elevated D-dimer levels have been reported in patients with breast, prostate, gynecologic, and lung cancers without clinical thrombosis [9]. As early as 1991, Mitter reported elevated D-dimer levels in patients with breast cancer [10].

In recent years, growing evidence has suggested a connection between D-dimer levels and the underlying biology of breast cancer. Evidence from several studies indicates that breast cancer patients exhibit markedly higher plasma D-dimer levels relative to individuals with benign breast conditions or healthy subjects. This elevation may reflect activation of the coagulation system associated with tumor progression. Although D-dimer has been proposed as a complementary marker for breast cancer detection, its diagnostic utility requires cautious interpretation and further validation in prospective studies. Notably, several studies have reported that D-dimer may exhibit superior sensitivity and specificity for breast cancer detection compared to traditional tumor markers such as CA 15-3 and CEA [11].

Cancer cells that remain alive after CT may develop recurrence or metastasis after remaining dormant for years. One of the earliest indicators of this renewed activity may be disturbances in the body’s clotting system. Therefore, thrombotic biomarkers such as D-dimer could potentially reflect tumor-related coagulation activation and may be explored in future studies as candidate markers of recurrence risk [12].

Fibrin remodeling plays a key role in this process, as it supports angiogenesis, supplying blood flow to metastatic tumors for growth.

Several studies have reported an association between breast cancer and D-dimer levels. However, to our knowledge, no study has specifically demonstrated the prognostic significance of D-dimer exclusively in HR-positive breast cancer. Elevated D-dimer levels have been reported as a negative prognostic indicator in hormone receptor-negative breast cancer. A meta-analysis showed that D-dimer levels were associated with breast cancer, particularly in progesterone receptor-negative tumors, with no significant correlation observed with estrogen receptor status [13]. Another study evaluated D-dimer levels and ER/PR status separately and found that elevated D-dimer levels were associated with advanced disease stage, nodal positivity, and vascular invasion, but no direct significant association with ER or PR expression was identified [14].

In our study, the median D-dimer level was ≤0.3 µg in patients with early-stage, non-metastatic breast cancer and was significantly lower than that observed in patients with advanced or metastatic disease. The finding that survival rates were reduced in patients with D-dimer levels > 0.3 µg suggests that elevated D-dimer may be associated with worse prognosis even in HR-positive breast cancer.

Our findings demonstrate that elevated pre-treatment D-dimer levels are significantly associated with advanced disease features such as stage IV status, poor performance (ECOG > 2) and the presence of bone or brain metastases. Both univariate and multivariate analyses showed worse survival in patients with pre-RT D-dimer > 0.3 ng/mL, ECOG > 2. Recent studies have shown a clear link between circulating tumor cells and elevated plasma D-dimer levels in patients with metastatic breast cancer. During cancer progression, several coagulation-related factors, such as tissue factor, fibrin, and plasmin, become dysregulated. These changes play a role not only in tumor growth and metastasis but also in thrombosis and angiogenesis [15,16].

Elevated D-dimer levels serve as a significant indicator of lymphovascular invasion, lymph node involvement, and distant metastasis. In this study, we found that D-dimer levels were significantly elevated in patients with more advanced disease. D-dimer was higher among those with distant metastases compared to non-metastatic patients, and it increased progressively with TNM stage. Furthermore, D-dimer was influenced by treatment context: for instance, patients undergoing systemic therapy for metastatic disease or those receiving larger radiation fields tended to exhibit higher levels, likely reflecting tumor burden and disease aggressiveness [13].

This study also revealed that patients who underwent CT exhibited higher D-dimer levels, indicating that treatment modalities may influence coagulation status, potentially reflecting tumor dynamics [7]. Overall prognosis was significantly better in patients who received chemotherapy.

Our study shows that baseline D-dimer levels were significantly associated with survival outcomes. Patients with D-dimer levels exceeding 0.65 ng/mL had significantly poorer OS and PFS in univariate and multivariate analyses. Analyses showed that median OS and PFS were 25.5 and 12.5 months, respectively, when D-dimer > 0.65 μg/mL.

Such findings are consistent with earlier research that associates D-dimer with cancer-related outcomes; for instance, a substantial prospective investigation revealed that individuals positioned within the uppermost quartile of D-dimer had significantly reduced OS in comparison to those exhibiting lower levels of D-dimer [17].

These results are consistent with previous and more recent evidence demonstrating that elevated D-dimer concentrations correlate with tumor burden, faster disease progression, and reduced overall survival in metastatic breast cancer patients. These results are consistent with previous studies highlighting the correlation between D-dimer concentrations and tumor burden, disease progression rates, and overall survival among breast cancer patients with metastasis [18]. In a comparable manner, Gomez-Rosas et al. reported on the prognostic efficacy of coagulation-related biomarkers, including D-dimer, in forecasting early disease recurrence within high-risk populations in breast cancer [19].

In advanced cancer, pro-inflammatory cytokines such as IL-6 are elevated and can initiate the coagulation cascade by upregulating tissue factor expression, resulting in increased D-dimer concentrations. In parallel, IL-6 stimulates the liver to synthesize CRP, another biomarker that escalates in response to aggressive disease. Clinically, patients presenting with elevated levels of IL-6 and CRP often demonstrate concurrent increases in D-dimer levels, and this combination is correlated with poor clinical outcomes [20].

Elevated CRP and IL-6 levels were associated with particularly poorer OS and PFS, consistent with prior findings linking high D dimer concentrations to unfavorable survival outcomes in breast cancer [21].

In this study, a significant moderate positive correlation was observed between D-dimer levels and lymphocyte percentage, as well as an inverse correlation for IL-6 levels. Patients with lymphocyte levels ≤ 15% had a median OS and PFS of 21 and 12 months, respectively, compared to 60 months for both OS and PFS in those with lymphocyte levels > 15%.

Likewise, patients with IL-6 levels > 7 pg/mL had a median OS and PFS of 27 and 19 months, respectively, while those with IL-6 levels ≤ 7 pg/mL demonstrated median OS and PFS of 60 months each.

Clinical studies indicate that peripheral depleted lymphocyte counts are often correlated with tumor burden and disease stage in breast carcinoma. One study reported lymphopenia in circa 20% of the patients identified with advanced breast cancer, in stark contrast to only 3% among early-stage cases, thus emphasizing its role as a possible marker of disease severity [22,23].

D-dimer testing is widely available and cost-effective, making it suitable for routine clinical use. An elevated baseline D-dimer may indicate the potential presence of metastases or suggest an aggressive tumor phenotype, which may reflect a more aggressive disease phenotype and higher tumor burden. However, given the retrospective nature of our study, clinical recommendations regarding surveillance strategies or treatment intensification based solely on D-dimer levels cannot be made [24].

Similarly, longitudinal assessment of D-dimer during follow-up may be explored in future prospective trials as a potential adjunct to imaging and standard tumor biomarkers.

In this study, patients with low ER/PR expression (<20%) showed both higher median pre-RT D-dimer levels (median 1.35 µg) and shorter survival time compared to patients with high expression (>70%) (median 0.15 µg). Median survival time was 27.5 months in the ER/PR < 20% group and 49.5 months in the ER/PR > 70% group. These findings suggest that low ER/PR expression may be associated with poor prognosis.

Although elevated D-dimer levels have previously been associated with poor prognosis mainly in hormone receptor-negative breast cancer, evidence regarding their prognostic value in HR-positive disease has been limited. Our findings suggest that even in HR-positive breast cancer, elevated D-dimer levels are associated with inferior survival outcomes. Nevertheless, these results should be interpreted as hypothesis-generating. Prospective studies are warranted to validate these results and to clarify the potential role of D-dimer in risk stratification and follow-up strategies for HR-positive breast cancer.

Study limitations: Several constraints were identified in this investigation. This is a retrospective and single-center study, which may lead to selection bias and limit the applicability of our findings to various populations or healthcare settings. Although the sample size remains relatively limited, it was sufficient to demonstrate significant associations; validation in larger multi-center cohorts is necessary to confirm D-dimer’s prognostic value. The cohort included both adjuvant and palliative radiotherapy populations, which may introduce clinical heterogeneity. However, this design was intended to explore the prognostic relevance of baseline D-dimer independent of treatment intent, as D-dimer reflects systemic tumor–host interaction, coagulation activation, and overall disease burden across the disease spectrum.

## 5. Conclusions

In summary, elevated D-dimer levels were significantly associated with adverse clinicopathologic features and poorer survival outcomes in patients with breast cancer. Measurement of D-dimer is widely available and cost-effective; however, given the retrospective and single-center nature of this study, the results should be interpreted cautiously and considered hypothesis-generating. Prospective large-scale multi-center studies are required to validate these findings and to determine whether D-dimer can be reliably incorporated into routine prognostic assessment, risk stratification, follow-up strategies, or clinical decision-making, particularly in hormone receptor-positive breast cancer.

## Figures and Tables

**Table 1:** Patient characteristics.

Characteristics	N = 112
Age	
Mean ± S.D.	54 ± 12
Median (min_max)	51 (38_88)
Comorbidity, *n* (%)	
No	96 (86)
Yes	16 (14)
Histological type, *n* (%)	
Invasive ductal carcinoma	112 (100)
Metastasis location, *n* (%)	
Bone *	22 (20)
Brain *	9 (6.3)
None	81 (72.3)
Stage, *n* (%)	
I/II/III	81 (72.3)
IV	31 (27.6)
ECOG, *n* (%)	
≤2	95 (85)
>2	17 (15)
ER/PR receptor level, *n* (%)	
Low (5–20%)	23 (20.5)
Moderate (30–60%)	20 (17.8)
High (70–100%)	69 (61.6)
Chemotherapy, *n* (%)	
Yes	83 (74.1)

*: Oligometastasis.

**Table 2:** RT characteristics of breast cancer patients.

Characteristics	*n* (%)
Palliative RT dose, *n* (%)	
1600–4000 cGy (2–16 fract.)	31 (27.6)
Adjuvant RT dose, *n* (%)	
4250–6000 cGy (15–30 fract.)	81 (72.3)
RT fract dose	
Adjuvant RT	
200–270 cGy	81 (72.3)
Palliative RT	
250–900 cGy	31 (27.6)
RT location, *n* (%)	
Adjuvant RT	81 (72.3)
Breast	41 (36.6)
Breast + axilla + level 1–3 supra	15 (13.9)
Breast + axilla + level 3	8 (7.1)
Chest wall + axilla + level 1–3 supra	17 (15.2)
Palliative RT	31 (27.6)
Bone *	22 (19.6)
Brain *	9 (8)

*: Oligometastasis. Supra: supraclavicular lymph nodes.

**Table 3:** Comparison of D-dimer levels before and after RT.

	Before RT N = 112 ^1^	After RT N = 112 ^1^	*p*-Value ^2^
D-dimer, µg/mL	0.37 (0.10–14.00)	0.32 (0.10–16.00)	0.076

^1^ Median (min–max). ^2^ Wilcoxon signed-rank test with continuity correction.

**Table 4:** D-dimer levels before and after RT for clinical characteristics.

	Before RT		After RT	
	D-Dimer, µg/mL ^1^	*p*-Value ^2^	D-Dimer, µg/mL ^1^	*p*-Value ^2^
Comorbidity		0.499		0.309
No	0.31 (0.10–14.00)		0.30 (0.10–16.00)	
Yes	0.45 (0.10–5.90)		0.43 (0.10–5.90)	
Metastasis location		**<0.001**		**<0.001**
Bone *	1.80 (0.10–14.00)		1.55 (0.10–16.00)	
Brain *	1.50 (0.32–2.40)		1.50 (0.40–2.30)	
None	0.30 (0.10–5.90)		0.30 (0.10–6.10)	
Stage		**<0.001**		**<0.001**
I/II/III	0.30 (0.10–5.90)		0.30 (0.10–6.10)	
IV	1.80 (0.10–14.00)		1.50 (0.10–16.00)	
ECOG		**<0.001**		**<0.001**
≤2	0.30 (0.10–14.00)		0.30 (0.10–16.00)	
>2	1.90 (0.10–4.60)		1.60 (0.10–3.00)	
ER/PR receptor level		**0.011**		**<0.001**
Low (10–20%)	0.50 (0.10–3.00)		0.50 (0.10–3.00)	
Moderate (30–60%)	0.60 (0.10–5.90)		0.60 (0.10–6.10)	
High (70–100%)	0.30 (0.10–14.00)		0.20 (0.10–16.00)	
RT location		**<0.001**		**<0.001**
Breast/chest wall	0.30 (0.10–5.90)		0.30 (0.10–6.10)	
Bone *	1.85 (0.10–14.00)		1.65 (0.10–16.00)	
Brain *	1.00 (0.32–2.40)		1.14 (0.40–2.30)	
RT fraction dose		**<0.001**		**<0.001**
200–270 cGy	0.30 (0.10–5.90)		0.30 (0.10–6.10)	
250–900 cGy	1.80 (0.10–14.00)		1.55 (0.10–16.00)	
Total RT dose		**<0.001**		**<0.001**
2500–4000 cGy	1.06 (0.10–14.00)		0.79 (0.10–16.00)	
4250–6000 cGy	0.30 (0.10–3.00)		0.30 (0.10–3.00)	
CT		**0.043**		**0.019**
No	0.20 (0.10–5.90)		0.20 (0.10–6.10)	
Yes	0.40 (0.10–14.00)		0.40 (0.10–16.00)	
Survival		**<0.001**		**<0.001**
Alive	0.30 (0.10–5.90)		0.30 (0.10–6.10)	
Dead	1.90 (0.30–14.00)		1.60 (0.40–16.00)	

Note: Significant *p*-values and rho coefficients are indicated in bold. *: Oligometastatic. ^1^ Median (min–max). ^2^ Wilcoxon rank-sum test; Kruskal–Wallis rank-sum test.

**Table 5:** Correlations between clinical parameters and D-dimer levels before and after RT.

Parameters	D-Dimer Before RT		D-Dimer After RT	
	D-Dimer, µg/mL ^1^	*p*-Value ^2^	D-Dimer, µg/mL ^1^	*p*-Value ^2^
Age	r = 0.11 (*p* = 0.229)		r = 0.13 (*p* = 0.184)	
Hematocrit	r = −0.072 (*p* = 0.454)		r = −0.03 (*p* = 0.752)	
Lymphocyte percentage	**r = −0.55 (*p* < 0.001)**		**r = −0.54 (*p* < 0.001)**	
Platelet count	**r = −0.016 (*p* = 0.866)**		**r = −0.018 (*p* = 0.853)**	
CRP	**r = 0.6 (*p* < 0.001)**		**r = 0.59 (*p* < 0.001)**	
Ferritin	**r = 0.42 (*p* < 0.001)**		**r = 0.41 (*p* < 0.001)**	
IL-6	**r = 0.72 (*p* < 0.001)**		**r = 0.68 (*p* < 0.001)**	
Fibrinogen	**r = 0.61 (*p* < 0.001)**		**r = 0.57 (*p* < 0.001)**	
INR	**r = −0.51 (*p* < 0.001)**		**r = −0.53 (*p* < 0.001)**	

^1^ D-Dimer values are expressed in micrograms per milliliter (µg/mL). ^2^ *p*-value indicates the statistical significance of the comparison. Note: Significant *p*-values and rho coefficients are indicated in bold.

**Table 6:** Univariate and multivariate analysis for OS.

Variable	Univariate		Multivariate	
	HR (95% CI)	*p*-Value	HR (95% CI)	*p*-Value
Age <45/≥45	0.13 (0.04–0.41)	**<0.001**	0.28 (0.09–0.86)	**0.02**
Stage IV disease	14.3 (5.26–39.1)	**<0.001**	4.8 (1.9–12.2)	**0.002**
ECOG > 2	9.11 (3.94–21.1)	**<0.001**	5.21 (1.55–17.6)	**0.008**
Metastatic site				
Bone	12.1 (4.47–33.0)	**<0.001**	3.6 (1.4–9.1)	**0.01**
Brain	13.8 (4.19–45.3)	**<0.001**	3.7 (1.4–9.8)	**0.01**
ER/PR receptor level				
≥70%/<20%	0.27 (0.15–0.67)	**<0.001**	0.50 (0.27–0.92)	**0.03**
Lymph > 15%/≤15%	0.05 (0.02–0.12)	**<0.001**	0.08 (0.02–0.30)	**<0.001**
IL-6 > 7/≤7 pg/mL	9.39 (3.81–23.1)	**<0.001**	0.79 (0.22–2.79)	0.711
Adj RT/palliative RT	0.15 (0.06–0.41)	**<0.001**	0.30 (0.06–0.92)	**0.048**
CT received/none	0.5 (0.15–0.99)	**0.049**	0.7 (0.25–1.3)	**0.18**
Pre-RT D-dimer > 0.3 µg/mL	6.13 (2.45–12.26)	**<0.001**	4.55 (1.89–11.3)	**0.002**
Pre-RT D-dimer > 0.5 µg/mL	8.45 (3.21–19.73)	**<0.001**	4.37 (1.72–9.86)	**0.002**
Pre-RT D-dimer > 0.65 µg/mL	10.6 (3.9–28.7)	**<0.001**	5.1 (1.9–13.6)	**<0.001**

CI = Confidence Interval; HR = Hazard Ratio; CT = chemotherapy; Lymph = lymphocyte; Adj = adjuvant. Significant *p*-values are indicated in bold.

**Table 7:** Univariate and multivariate analysis for PFS.

Variable	Univariate		Multivariate	
	HR (95% CI)	*p*-Value	HR (95% CI)	*p*-Value
Age < 45/≥45	0.22 (0.093–0.56)	**0.001**	0.42 (0.16–0.98)	**0.045**
Stage IV/I disease	9.6 (4.14–22.3)	**<0.001**	3.9 (1.68–9.4)	**0.003**
ECOG > 2/≤2	7.4 (3.2–17.1)	**<0.001**	4.2 (1.4–12.6)	**0.01**
Metastatic site				
Bone	8.73 (3.52–21.6)	**<0.001**	3.16 (1.38–7.6)	**0.01**
Brain	9.91 (3.75–26.1)	**<0.001**	3.4 (1.33–8.8)	**0.01**
ER/PR receptor				
≥70%/<20%	0.38 (0.23–0.74)	**0.003**	0.58 (0.31–0.96)	**0.04**
Lymph > 15%/≤15%	0.09 (0.04–0.20)	**<0.001**	0.14 (0.05–0.38)	**<0.001**
IL-6 > 7/≤7 pg/mL	6.29 (2.86–13.81)	**<0.001**	1.12 (0.4–3.36)	0.86
Adj RT/palliative RT	0.26 (0.11–0.58)	**0.001**	0.41 (0.18–0.95)	**0.038**
CT received/none	0.63 (0.32–0.99)	**0.048**	0.78 (0.35–1.6)	0.42
Pre-RT D-dimer > 0.3 µg/mL	4.9 (2.32–10.17)	**<0.001**	3.43 (1.54–7.8)	**0.004**
Pre-RT D-dimer > 0.5 µg/mL	6.5 (2.9–14.6)	**<0.001**	3.88 (1.67–9.1)	**0.003**
Pre-RT D-dimer > 0.65 µg/mL	8.95 (3.62–22.1)	**<0.001**	4.68 (1.83–11.9)	**0.002**

Significant *p*-values are indicated in bold.

**Table 8:** Association of ER/PR expression (>70 vs. <20) with D-dimer levels and OS.

ER/PR	Patient Number (*n*)	Median D-Dimer (µg)	Median OS (Months)	*p*-Value
>70%	73	0.15	49.5	
<20%	39	1.36	27.5	**0.002**

Variables were compared using the Mann–Whitney U test and are presented as median values. ER/PR: estrogen/progesterone receptor. Significant *p*-values are indicated in bold.

**Table 9:** Association of ER/PR expression (>70 vs. <20) with D-dimer levels and PFS.

ER/PR	Patient Number (*n*)	Median D-Dimer (µg)	Median PFS (Months)	*p*-Value
>70%	73	0.15	49.5	
<20%	39	1.36	23.0	**0.002**

Variables were compared using the Mann–Whitney U test and are presented as median values. Significant *p*-values are indicated in bold.

## Data Availability

The datasets analyzed during the current study are not publicly available due to institutional policy but are available from the corresponding author on reasonable request.

## Abbreviations

The following abbreviations are used in this manuscript:

ER	Estrogen receptor
PR	Progesterone receptor
HR	Hormone receptor
PFS	Progression-free survival
OS	Overall survival
RT	Radiotherapy
CT	Chemotherapy
ECOG	Eastern Cooperative Oncology Group
CRP	C-reactive protein
IL-6	Interleukin-6
INR	International normalized ratio
CI	Confidence Interval
HR	Hazard Ratio
Gy	Gray
CEA	Carcinoembryonic Antigen
